# Effect of phytic acid on bond strength and interfacial integrity of universal adhesive to deep dentin

**DOI:** 10.1590/0103-6440202204810

**Published:** 2022-10-21

**Authors:** Ahmed Mostafa Attia, Ahmed Fawzy Abo-Elezz, Rehab Khalil Safy

**Affiliations:** 1 Department of Conservative Dentistry, Faculty of Dentistry, Suez Canal University, Egypt,

**Keywords:** deep dentin, microtensile bond strength, phytic acid, phosphoric acid, universal adhesive

## Abstract

This study investigated the effect of phytic acid (IP6) in different concentrations and application times on microtensile bond strength (µTBS) and interfacial integrity of universal adhesive to deep dentin. Flat deep dentin surfaces of 48 human molars were either etched with 37% phosphoric acid (PA) for 15 sec (control); or received no acid treatment and universal adhesive was applied directly in a self-etch mode (SE); or divided according to IP6 concentration (C) into two main groups: C1, 0.5%, and C2, 1%. Specimens of IP6 groups were further subdivided into three subgroups according to application time of IP6 (T) where; T1, 15 sec; T2, 30 sec and T3, 60 sec. Single Bond Universal Adhesive was then applied and resin composite blocks were built-up. Forty Specimens were then sectioned to produce resin/dentin beams that were used for µTBS testing using a universal testing machine. The remaining eight specimens were sectioned into slabs that were immersed into ammonical silver nitrate solution and nanoleakage was observed using a field emission scanning electron microscope (FE-SEM). The results showed that the application of IP6 in 0.5% and 1% produced significantly higher µTBS and less nanoleakage compared to PA and SE groups. Also, the application of IP6 for 60 sec recorded the highest µTBS and the lowest nanoleakage followed by 30 sec, and 15 sec respectively. Therefore, conditioning of deep dentin with IP6 enhances µTBS and interfacial integrity of universal adhesive to deep dentin in comparison to PA etching or using the universal adhesive in SE mode.

## Resumo

Este estudo investigou o efeito do ácido fítico (IP6) em diferentes concentrações e tempos de aplicação na resistência da ligação microtensiva (µTBS) e na integridade interfacial do adesivo universal à dentina profunda. As superfícies planas de dentina profunda de 48 molares humanos foram preparadas com ácido fosfórico (PA) a 37% durante 15 seg. (controlo); ou não receberam tratamento ácido e o adesivo universal foi aplicado diretamente em modo de auto-adesivo (SE); ou dividido de acordo com a concentração de IP6 (C) em dois grupos principais: C1, 0,5%, e C2, 1%. Os espécimes dos grupos IP6 foram ainda subdivididos em três subgrupos de acordo com o tempo de aplicação do IP6 (T) em que; T1, 15 seg; T2, 30 seg e T3, 60 seg. Aplicou-se então Single Bond Universal Adhesive e construíram-se blocos compostos de resina. Quarenta espécimes foram então seccionados para produzir feixes de resina/dentina que foram usados para testes µTBS usando uma máquina de testes universal. Os restantes oito espécimes foram seccionados em placas que foram imersas em solução amoniacal de nitrato de prata e observou-se uma nano-infiltração utilizando um microscópio eletrônico de varrimento de emissões de campo (FE-SEM). Os resultados mostraram que a aplicação de IP6 em 0,5% e 1% produziu µTBS significativamente mais elevado e menos nano-infiltração em comparação com os grupos PA e SE. Além disso, a aplicação de IP6 durante 60 seg registou o µTBS mais alto e o mais baixo de nano-infiltração seguido de 30 seg, e 15 seg respectivamente. Portanto, o condicionamento da dentina profunda com IP6 melhora o µTBS e a integridade interfacial do adesivo universal à dentina profunda em comparação com a gravação de PA ou utilizando o adesivo universal no modo SE.

## Introduction

Resin composite bond strength to dentin has an important role in restoration success. Bonding mechanism of dentin is different from enamel due to basic differences in their organic and inorganic material ^1^. Dental enamel is highly mineralized tissue composed of 85 vol% minerals and a low percentage of protein, lipid and water ^2^ while dentin is composed of approximately 50 vol% mineral phase, 30 vol% collagen and 20 vol% water ^3^. Therefore, bonding to dentin represents a much greater challenge than to enamel ^4^ especially in deeper dentin due to the decrease in inter-tubular dentin and the increase in tubular diameter as well as water content ^5^.

Over the years, phosphoric acid (PA) etching has been used to enhance the substrate surface characteristics before the application of the adhesive system ^6^. Dentin etching with phosphoric acid is performed to remove the smear layer ^7^ ^)^ and expose the collagen network that will be later infiltrated by the adhesive forming the hybrid layer ^8^. However, phosphoric acid creates a delicate collagen network that is depleted of hydroxyapatite and that would collapse upon drying preventing the effective infiltration of the adhesive ^9^ ^,^ ^10^.

 Recently, an adhesive categorized as “universal” or “multi-mode”, as it can be used in both etch-and-rinse and self-etch modes, has been introduced ^11^. Different monomers and primers such as silane and 10-methacryloyloxydecyldihydrogen phosphate (MDP) are incorporated in some universal adhesive systems to improve their bonding effectiveness to the tooth structure ^12^. The bond strength of universal adhesives was acceptable with different etching protocols ^13^; however, there is still debate about the ideal mode of application of universal adhesives, whether etch-and-rinse or self-etch ^14^.

Phytic acid (IP6) is an organic acid present in our daily diet including cereals, legumes, oilseeds and nuts ^15^. It is a highly negatively charged molecule making it an effective chelator for multivalent cations such as calcium (Ca^+2^) ^16^. Although it is currently used for removal of smear layer in endodontics ^17^, a limited number of studies were concerned about its use in improvement of bond strength to coronal dentin.

Therefore, the aim of this study was to investigate the effect of different concentrations and application times of IP6 on µTBS and nanoleakage of universal adhesive to deep dentin in comparison to PA etching or using the universal adhesive in SE mode. The tested null hypothesis was that IP6 had no significant effect on µTBS and nanoleakage to deep dentin.

## Materials and Methods

The materials used in this study are described in Box 1.

**Box 1:**
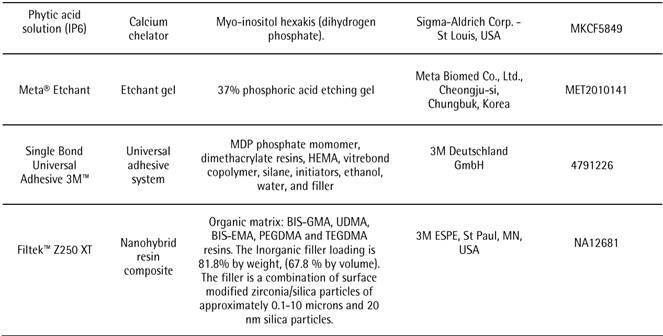
Materials’ description, composition, manufacturers and lot numbers.

*Abbreviations:* MDP, 10-methacryloyloxydecyl dihydrogen phosphate; HEMA, 2-hydroxyethyl methacrylate; BIS-GMA, Bisphenol A-diglycidyl dimethacrylate; UDMA, Urethane Dimethacrylate; BIS EMA, Bisphenol A-polyethylene glycol diether dimethacrylate; PEGDMA, polyethylene glycol dimethacrylate; TEGDMA, Triethylene glycol dimethacrylate.

### Selection of teeth

A total of 48 intact, non-carious, freshly permanent human molar teeth extracted from 20- to 40- years old individuals for periodontal reasons were used in this study for preparation of specimens for microtensile bond strength and nanoleakage tests. The sample size was calculated using G*Power software version 3.1.9.2 to detect the effect size of 0.25, a power of 85% and at a significant level of 5% (p<0.05) and a partial eta squared of 0.06. Teeth were selected according to the protocol approved by the Ethical Committee of the Faculty of Dentistry, Suez Canal University, Egypt (number 170/2019). Immediately after extraction, teeth were thoroughly washed under running water to remove blood and mucous, scaled to remove calculus and remnants of periodontal ligament tissue and polished with fine pumice and soft rubber cups at conventional speed. Teeth were examined for freedom of cracks using a magnifying lens (5x). All teeth exhibiting any signs of caries, microcracks or any other defective structure were discarded. The collected teeth were stored in normal saline plus 0.5% thymol until being used for no longer than 3 months ^18^.

### Preparation of specimens

Teeth were fixed in acrylic resin blocks then mounted in an automated diamond saw (Isomet 4000, Buehler Ltd., Lake Bluff, USA), which was used for all sectioning procedures in this study. The occlusal enamel of all teeth was removed using diamond disc under water cooling to expose superficial dentin just beneath the central groove. Then, with size 2 (ADA size number) round carbide bur (DIA TESSIN, Switzerland), an indentation of 2mm depth in dentin was prepared. The depth of the indentation was guided using a rubber stopper mounted to the shaft of the round bur. Deep dentin was reached by removal of the occlusal surface with the same diamond disc under water cooling till the indentation disappears ^19^. The exposed dentin surfaces were then polished with wet 600-grit SiC abrasive paper for 60 seconds to create a standardized smear layer.

### Microtensile bond strength (μTBS) testing

Forty specimens were used for the microtensile bond strength testing. Five specimens were used as a control group (n=5), where dentin surfaces were etched with 37% PA (Meta Biomed Co., Ltd., Cheongju-si, Chungbuk, Korea) for 15 sec then rinsed for 20 sec and blot-dried with a moist cotton pellet. Another five specimens didn’t receive any acid pre-treatment and the universal adhesive was applied in a self-etch mode (SE) (n=5). The remaining 30 specimens were divided according to concentration of IP6 solution (C) (Sigma-Aldrich Corp., St Louis, USA) into two main groups (n=15): C1 group, dentin surfaces were conditioned with 0.5% IP6 and C2 group, dentin surfaces were conditioned with 1% IP6. Specimens of IP6 groups were further subdivided into three subgroups (n=5) according to application time of IP6 solution (T) as follow: subgroup T1, 15 sec; subgroup T2, 30 sec and subgroup T3, 60 sec. IP6 solution was applied concerning its concentrations and application times using a plastic syringe and then dentin surfaces were rinsed for 10 sec and blot-dried.

The adhesive system (Single Bond Universal, 3M Deutschland GmbH) was applied to the exposed dentin surfaces according to manufacturer’s instructions then light-cured for 10 sec using a LED light curing unit (Elipar S10, 3MESPE, St Paul, MN, USA, light intensity 1200 mW/cm^2^). Nano-hybrid resin composite (Filtek™ Z250 XT, 3M ESPE) was then incrementally packed in two horizontal layers of 2 mm thickness to form resin composite blocks of 4 mm height using a teflon mold (8 x 8 x 4 mm). Each increment was light-cured for 10 sec according to manufacturer’s instructions at a right angle from the occlusal surface then all specimens were stored in distilled water at room temperature for 24 h.

### Microtensile bond strength (μTBS) measurement

Specimens were mounted in a specially designed gripping attachment and serially sectioned in bucco-lingual direction to obtain slabs, then further sectioned to obtain (0.9 mm x 0.9 mm) resin composite/dentin beams ^20^. The resultant beams were individually measured with a digital caliper and attached to geraldeli’s jig ^21^ using cyanoacrylate glue (Zapit Dental Ventures of America Inc., Corona, CA, USA) ^22^ full stop. The jig was in turn mounted into a universal testing machine (Instron, MA, USA) with a load cell of 500 N at a cross-head speed of 0.5 mm/min and stressed under tension until failure. The μTBS was calculated in MegaPascal (MPa) by a software (Bluehill Lite software, England).

### Nanoleakage evaluation

Eight specimens were used for nanoleakage evaluation, where one specimen was used for PA group (control) and one specimen was used for SE group. The remaining six specimens were divided according to concentration of IP6 into two main groups (n=3) and each group was further subdivided according to application time of IP6 (n=1) as mentioned before in μTBS testing. Specimens were stored in distilled water at room temperature for 24 h then were sectioned into slabs (approximately 1 mm-thick) by slow-speed diamond saw. One central slab were chosen from each tooth ^23^ that were then coated with two layers of fast-drying nail varnish (Colorama, CEIL) away from the resin /dentin interface by 1 mm on each side ^24^. The prepared slabs were immersed in 50 % (W/V) ammonical silver nitrate (AgNO3) tracer solution for 24 h in black photo-film containers to ensure total darkness. Slabs were then rinsed with distilled water and immersed in a photo-developing solution (Kodak Professional D-76 developer, Kodak Rochester, NY) for 8 h under the effect of fluorescent light ^25^. Slabs were then wet-polished using 2000-grit SiC paper and diamond pastes (Buehler Ltd., Lake Bluff, IL, USA) ^26^, mounted on aluminum stubs and sputter-coated with gold (Edwards S150A, UK). Later on, slabs were observed using a field emission scanning electron microscope (FE-SEM) (QUANTA FEG 250) in backscattered electron mode with 20 kV and standardized 3000x magnification ^27^. Photomicrographs were obtained from each slab and silver nitrate uptake was evaluated using open-source Image J software (Image J, National Institute of Health, Bethesda, MD, USA).

### Statistical analysis

The mean and standard deviation values were calculated for each group in each test. Data were explored for normality using Kolmogorov-Smirnov and Shapiro-Wilk tests, data showed parametric (normal) distribution. One-way ANOVA followed by Tukey post-hoc test was used to compare between more than two groups in non-related samples. Also, Two-way ANOVA was used to test the interaction between variables. The significance level was set at P ≤ 0.05. Statistical analysis was performed with IBM® SPSS® Statistics Version 20 for Windows.

## Results

### Microtensile bond strength results

The mean and standard deviation of μTBS values of the tested groups are presented in Table 1. The results showed that IP6 groups (0.5% and 1%) had significantly higher μTBS mean values compared to PA group (control) and SE group (p<0.001). There was no statistically significant difference between PA group and SE group (p>0.001). Regarding C1 group, the experimental subgroup C1T3 recorded the highest μTBS mean value followed by C1T2, while the least μTBS mean value was found in C1T1, with a statistically significant difference only between subgroups C1T1 and C1T3 (p<0.001). Regarding C2 group, the experimental subgroup C2T3 showed the highest μTBS mean value followed by C2T2, while the least μTBS mean value was found in C2T1. A statistically significant difference was found between C2T1 and each of C2T2 and C2T3 subgroups (p<0.001).

**Table 1:** Mean and standard deviation (SD) values of microtensile bond strength (μTBS) in MPa for each tested group

Group	Microtensile bond strength (MPa)	
	Mean	SD
PA (control)	20.85 ^e^	4.28
SE (no acid pre-treatment)	18.80 ^e^	3.68
C1T1 (0.5% IP6/ 15 sec)	29.36 ^cd^	5.61
C1T2 (0.5% IP6/ 30 sec)	34.09 ^abc^	5.47
C1T3 (0.5% IP6/ 60 sec)	38.37 ^a^	4.68
C2T1 (1% IP6/ 15 sec)	26.03 ^d^	5.20
C2T2 (1% IP6/ 30 sec)	31.42 ^bc^	6.96
C2T3 (1% IP6/ 60 sec)	35.21 ^ab^	5.41
*p-value*	<0.001*	

Means with different letters in the same column indicate statistically significance difference. *; significant (p<0.05).

### Nanoleakage results

**Figure 1:**
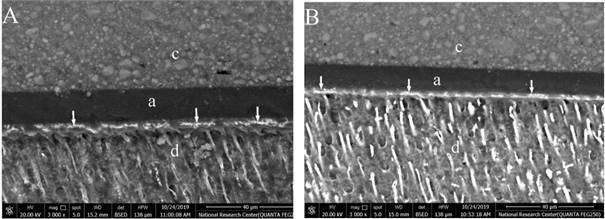
Representative FE-SEM photomicrographs of at (3000X) showing nanoleakage at the hybrid layer. (A) PA group (control), (B) SE group. The white arrows represent silver nitrate uptake, (c) composite, (a) adhesive and (d) dentin.

**Figure 2:**
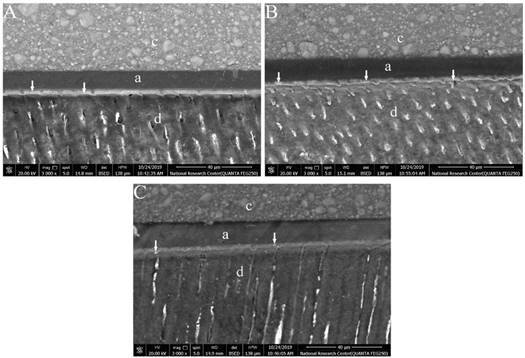
Representative FE-SEM photomicrographs at (3000X) showing nanoleakage at the hybrid layer. (A) Subgroup C1T1, (B) Subgroup C1T2 and (C) Subgroup C1T3. The white arrows represent silver nitrate uptake, (c) composite, (a) adhesive and (d) dentin.

**Figure 3:**
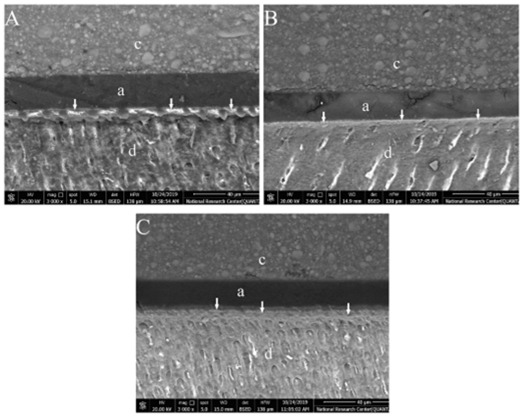
Representative FE-SEM photomicrographs at (3000X) showing nanoleakage at the hybrid layer. (A) Subgroup C2T1, (B) Subgroup C2T2 and (C) Subgroup C2T3. The white arrows represent silver nitrate uptake, (c) composite, (a) adhesive and (d) dentin.

FE-SEM photomicrographs showed different nanoleakage levels between groups. A distinctive silver-reticular pattern formation could be recognized along the hybrid layer of both PA and SE groups (Figure 1A and B) representing higher levels of nanoleakage. IP6 groups’ showed a decrease in nanoleakage levels along the hybrid layer that was represented by decrease in the thickness of the silver-reticular pattern as shown in the experimental subgroups C1T1, C1T2, C2T1 and C2T2 (Figure 2A, 2B, 3A & 3B). Increasing application time to 60 sec in both concentrations of IP6 resulted in distinct decrease in silver uptake that appears as spotted-pattern along the hybrid layer as shown in the experimental subgroups C1T3 and C2T3 (Figure 2C and 3C).

## Discussion

Deep dentin is much more difficult to bond compared to superficial dentin due to its low contents of intertubular dentin as well as higher amount of water as reported in some studies ^28^ ^,^ ^29^. Phosphoric acid has been widely used at concentrations of 35-37% for 15 sec for dentin etching ^30^. With these high concentrations, this acid creates a deep demineralized layer that may not be completely infiltrated by resin monomers ^31^. This layer of demineralized dentin is left unprotected by resin forming a weak zone of the bonded interface ^32^. Adhesive systems named “universal” or “multimode” were introduced to promote adhesion to dentin ^33^. Single Bond Universal Adhesive was used in the current study which contains 10-MDP functional monomer which chemically interacts with hydroxyapatite forming a stable nanolayer that could constitute a strong phase at the adhesive interface ^34^. Unfortunately, the lower calcium content which is needed for chemical bond with 10-MDP in addition to the increased dentin permeability in deep dentin may resulted in low bond strength values in deep dentin ^35^. Phytic acid is an organic acid found in cereals and legumes ^15^ and it was proved to be an effective agent in removing smear layer in endodontic treatment ^17^. It has a strong negative charge that can chelate positively charged multivalent cations of hydroxyapatite such as calcium (Ca^+2^) to form Ca-phytate salt which is characterized by very low solubility ^36^ ^,^ ^37^.

The results of this study showed that application of IP6 at both concentrations (0.5 % and 1%) in groups C1 & C2 respectively recorded significantly higher μTBS mean values and less nanoleakage levels than PA group. This result was confirmed by its FE-SEM photomicrographs which showed a thinner reticular pattern of silver uptake along the hybrid layer of IP6 groups’ than that of PA group. This result could be attributed to that PA etching causes deep demineralization that jeopardizes complete infiltration of resin monomers, thereby resulting in the formation of a weaker and unprotected demineralized dentin zone at the base of the hybrid layer ^38^. This poor impregnation of resin could result in low bond strength ^39^.

The results also revealed that IP6 groups’ recorded significantly higher μTBS mean values than SE group. In consistence with this result, FE-SEM photomicrographs showed a thicker silver-reticular pattern of nanoleakage along the hybrid layer of SE group in comparison to IP6 groups’. This result could be attributed to the ultra-mild acidity of Single Bond Universal Adhesive (pH 2.7) which resulted in incomplete penetration of acidic monomers into the smear layer and its partial dissolution and consequent minimal penetration into dentin beneath the smear layer ^40^. On the other hand, IP6 is more acidic (pH ≥ 1), so it has higher ability to remove the smear layer with a conditioning effect on the underlying dentin; this may have allowed better penetration of resin monomers into the demineralized dentin ^20^. IP6 also may function as a natural crosslinker to collagen fibrils of the demineralized dentin, making them more stable and less susceptible to collapse, resulting in better preservation of the interfibrillar spaces to enable better impregnation of monomers ^41^ ^,^ ^42^. The cross-linking effect of IP6 to collagen may be related to hydrogen bond that occurs between its hydroxyl groups and the amine groups of the side chain amino acid in collagen molecule ^43^. Additionally, IP6 can bind to protein and calcium to form a ternary complex (Protein-Ca-Phytate) which has a good chemical bond ^44^.

Regarding the effect of IP6 concentration, the results showed that C1group (0.5% IP6) recorded higher μTBS mean values and less nanoleakage levels than C2 group (1% IP6) group. This may be explained on the basis of that dentin conditioning with higher concentration of acids can result in excessive intertubular dentin demineralization and collagen collapse with suspected decrease in μTBS values ^45^.

Considering the effect of application time of IP6, the results revealed that increasing application time of IP6 had a positive effect on μTBS mean values to deep dentin. The results showed that the application of IP6 for 60 sec recorded significantly higher μTBS mean value than 30 sec and 15 sec respectively. Nanoleakage results also were in accordance with μTBS values, where increasing the conditioning time showed a distinct decrease in silver uptake along the hybrid layer from reticular-pattern to spotted-pattern as shown in FE-SEM photomicrographs. This result could be attributed to the fact that longer application time promote more dissolution of the smear layer on the dentin surface for better resin monomer diffusion, allowing improved mechanical interlocking and consequently higher resin-dentin µTBS ^46^.

In the present study, the use of IP6 to condition deep dentin surface significantly improved µTBS and nanoleakage of universal adhesive in comparison to phosphoric acid etching or using the universal adhesive in a self-etch mode. Thus, the null hypothesis was rejected.

## Recommendations

Further in vitro studies are needed to assess the effect of aging on microtensile bond strength and interfacial integrity of universal adhesive to deep dentin after conditioning with phytic acid.

Further in vivo studies are needed to assess the effect of phytic acid on microtensile bond strength and interfacial integrity of universal adhesive to deep dentin.

It is recommended to supply phytic acid in gel form for better manipulation than liquid form.

## Conclusions

Under the limitations of this *in vitro* study, the following could be concluded:

Conditioning of deep dentin with phytic acid (either 0.5% or 1%) enhance microtensile bond strength and interfacial integrity of universal adhesive to deep dentin in comparison to phosphoric acid etching or using the universal adhesive in a self-etch mode.

Increasing application time of phytic acid improves microtensile bond strength as well as interfacial integrity of universal adhesive to deep dentin.
